# Biomarkers in Psychiatry: Concept, Definition, Types and Relevance to the Clinical Reality

**DOI:** 10.3389/fpsyt.2020.00432

**Published:** 2020-05-15

**Authors:** Maria Salud García-Gutiérrez, Francisco Navarrete, Francisco Sala, Ani Gasparyan, Amaya Austrich-Olivares, Jorge Manzanares

**Affiliations:** ^1^Instituto de Neurociencias, Universidad Miguel Hernández-CSIC, Alicante, Spain; ^2^Red Temática de Investigación Cooperativa en Salud (RETICS), Red de Trastornos Adictivos, Instituto de Salud Carlos III, MICINN and FEDER, Madrid, Spain

**Keywords:** biomarkers, neuropsychiatry, personalized medicine, lymphocytes, peripheral biomarkers, central biomarkers

## Abstract

During the last years, an extraordinary effort has been made to identify biomarkers as potential tools for improving prevention, diagnosis, drug response and drug development in psychiatric disorders. Contrary to other diseases, mental illnesses are classified by diagnostic categories with a broad variety list of symptoms. Consequently, patients diagnosed from the same psychiatric illness present a great heterogeneity in their clinical presentation. This fact together with the incomplete knowledge of the neurochemical alterations underlying mental disorders, contribute to the limited efficacy of current pharmacological options. In this respect, the identification of biomarkers in psychiatry is becoming essential to facilitate diagnosis through the developing of markers that allow to stratify groups within the syndrome, which in turn may lead to more focused treatment options. In order to shed light on this issue, this review summarizes the concept and types of biomarkers including an operational definition for therapeutic development. Besides, the advances in this field were summarized and sorted into five categories, which include genetics, transcriptomics, proteomics, metabolomics, and epigenetics. While promising results were achieved, there is a lack of biomarker investigations especially related to treatment response to psychiatric conditions. This review includes a final conclusion remarking the future challenges required to reach the goal of developing valid, reliable and broadly-usable biomarkers for psychiatric disorders and their treatment. The identification of factors predicting treatment response will reduce trial-and-error switches of medications facilitating the discovery of new effective treatments, being a crucial step towards the establishment of greater personalized medicine.

## Introduction

According to World Health Organization mental illness presented devastating rates of prevalence, mortality, morbidity and disability. Suffering a serious mental illness reduces average life expectancy in 13 to 32 years (1, 2). Aside from mortality, in most Western countries, mental disorders are the leading cause of disability, responsible for 30-40% of chronic sick leave and costing approximately a 4% of gross domestic product (3). Besides, for all types of mental illness, pharmacological treatment options are scarce and present limited efficacy. Several studies highlighted that, in terms of recovery and remission, current pharmacological interventions showed significant limitations. A series of effectiveness trials sponsored by the National Institute of Mental Health (NIMH) in USA provided relevant data in this regard. In CATIE (Clinical Antipsychotic Trials of Intervention Effectiveness) study, 74% of patients suffering from chronic schizophrenia (SCZ) experienced problems of treatment adherence within 18 months (4). In addition, in the Sequenced Treatment Alternatives to Relieve Depression (STAR*D) study only 31% of patients with major depressive disorder (MDD) were in remission after being treated with a selective serotonin reuptake inhibitor for a total of 14 weeks (5). An additional study carried out in patients diagnosed with bipolar disorder (BD) (STEP-BD, Systematic Treatment Enhancement Program for Bipolar Disorder) revealed that only 24% of patients experienced a remission of depression during 8 consecutive weeks, outcome similar to those observed in the vehicle group (6).

Several factors contribute to this clinical reality. On one hand, the heterogeneity/complexity of mental disorders. Patients suffering from a mental illness displayed several symptoms related with behavior, thinking, feelings and/or social interaction. To facilitate the diagnoses, mental disorders are classified by diagnostic categories with a broad variety list of symptoms according to the Diagnostic and Statistical Manual of Mental Disorders, Fifth Edition (DSM-IV), or International Statistical Classification of Diseases and Related Health Problems, Tenth Edition (ICD-11). Consequently, patients diagnosed with the same psychiatric illness present a great heterogeneity in their clinical presentation. In addition, several mental illnesses present symptoms in common, that can often make the diagnosis difficult.

On the other hand, psychiatric diseases present high comorbidity. Approximately 85%–90% of patients with depression also experience symptoms of anxiety, and vice versa (7, 8). Among schizophrenic patients, psychiatric comorbidities are common. Around 50% of patients suffer from depression and more than 47% have a lifetime diagnosis of comorbid substance use disorders (9–11). The simultaneous presence of two or more psychiatric diseases are associated with greater severity, worse response to the pharmacological treatment and have a greater risk of suicide than either condition alone.

Despite these sobering facts, progress in human brain research and the advent of new technologies, such as ‘omics’ technologies, offers the opportunity for change mental health treatment and outcomes in a near future. In this respect, the identification of biomarkers has become a new promising tool for guiding diagnosis, predicting clinical outcome and, therefore, improving the understanding of the pathophysiology of mental disorders. This review provides an overview about the current state of biomarkers in neuropsychiatry, with the ultimate aim of remarking some goals achieved up to date and the future challenges needed to develop valid, reliable and broadly-usable biomarkers for psychiatric disorders and their treatment. For this purpose, the review includes a definition of biomarker’s concept throughout history, describes the different types of biomarkers and their potential role in clinical practice, and emphasizes the samples and techniques commonly used. The role of ‘omics’ is described in greater detail due to its huge progress in the recent years. A final conclusion remarks the difficulties and limitations of current biomarkers strategies in neuropsychiatry and the future challenges needed to progress in this field.

## What Are Biomarkers? Evolution of Biomarkers Through History

During the last 50 years, the definition of biomarker has been modified according to scientific and clinical progress. The term “biomarker” was used for the first time in 1973 to indicate the presence or absence of biological material. However, the concept is older, referenced as a “biochemical marker” in 1949 (12) and “biological marker” in 1957 (13).

In 2000, the Biomarker Definition Working Group, supported by the U.S. National Institute of Health (NIH), defined a biomarker as “a characteristic that is objectively measured and evaluated as an indication of normal biological processes, pathogenic processes, or pharmacologic responses to a therapeutic intervention” (14). This definition has two major limitations. The first one lies in the fact that sometimes a biomarker is measured by subjective parameters. The second one is the fact that additional processes or responses beyond those covered by the definition are excluded.

In 2016, Fitzgerald and colleagues redefined the concept of biomarker as “a functional variant or quantitative index of a biological process that predicts or reflects the evolution of or predisposition to a disease or a response to a therapy” (15). Nevertheless, this description lacks the consideration of structural variants and qualitative index as potential biomarkers.

In order to harmonize the term of biomarker, the Food and Drug Administration (FDA) in collaboration with the NIH Joint Leadership Council convened the FDA-NIH Biomarker Working Group in 2016. This group simplified the biomarker definition being considered as “a defined characteristic that is measured as an indicator of normal biological processes, pathogenic processes or responses to an exposure or intervention” (16). This definition, clearer and more concise, defines a biomarker specifying its principal applications without any unnecessary complexity or contradictory information. Besides, to ensure its clinical use, a good biomarker should be measured with high reproducibility, present a sizeable signal-to-noise ratio and, more importantly, meet the condition of being modified in a dynamic and reliable way as the clinical condition progress. In addition, a biomarker should be accessible for its detection and measurement, as would be the case of a plasmatic parameter or a genetic marker, or being detected by histological or image/neuroimaging techniques (17).

### Types and Role of Biomarkers in the Clinical Practice

**Figure 1:**
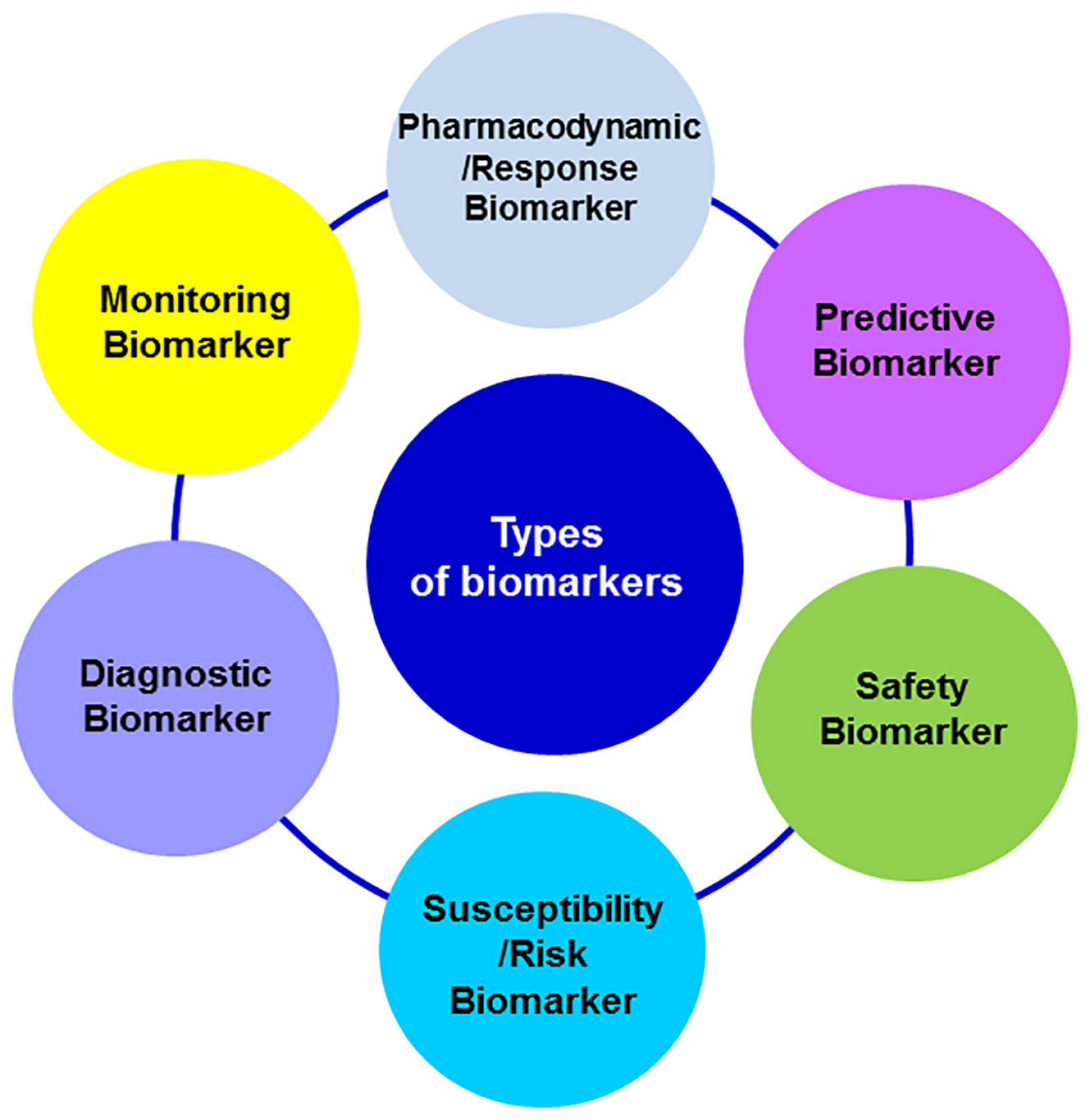
Classification of biomarkers based on its main clinical application.

According to their applications, biomarkers can provide complementary information about the disease or the intervention under consideration. Biomarkers may be identified at any event occurring since the pathogenesis, the onset of first clinical manifestations, diagnosis, treatment outcome or recovery. The FDA-NIH Biomarker Working Group distinguished several types of biomarkers based on their main clinical application: diagnostic, monitoring, pharmacodynamic/response, predictive r, prognostic, safety, and susceptibility/risk biomarkers (**Figure 1**). A biomarker may meet multiple criteria for different uses or present specific features that enable its particular use (18).

### Diagnostic Biomarker

**Figure 2:**
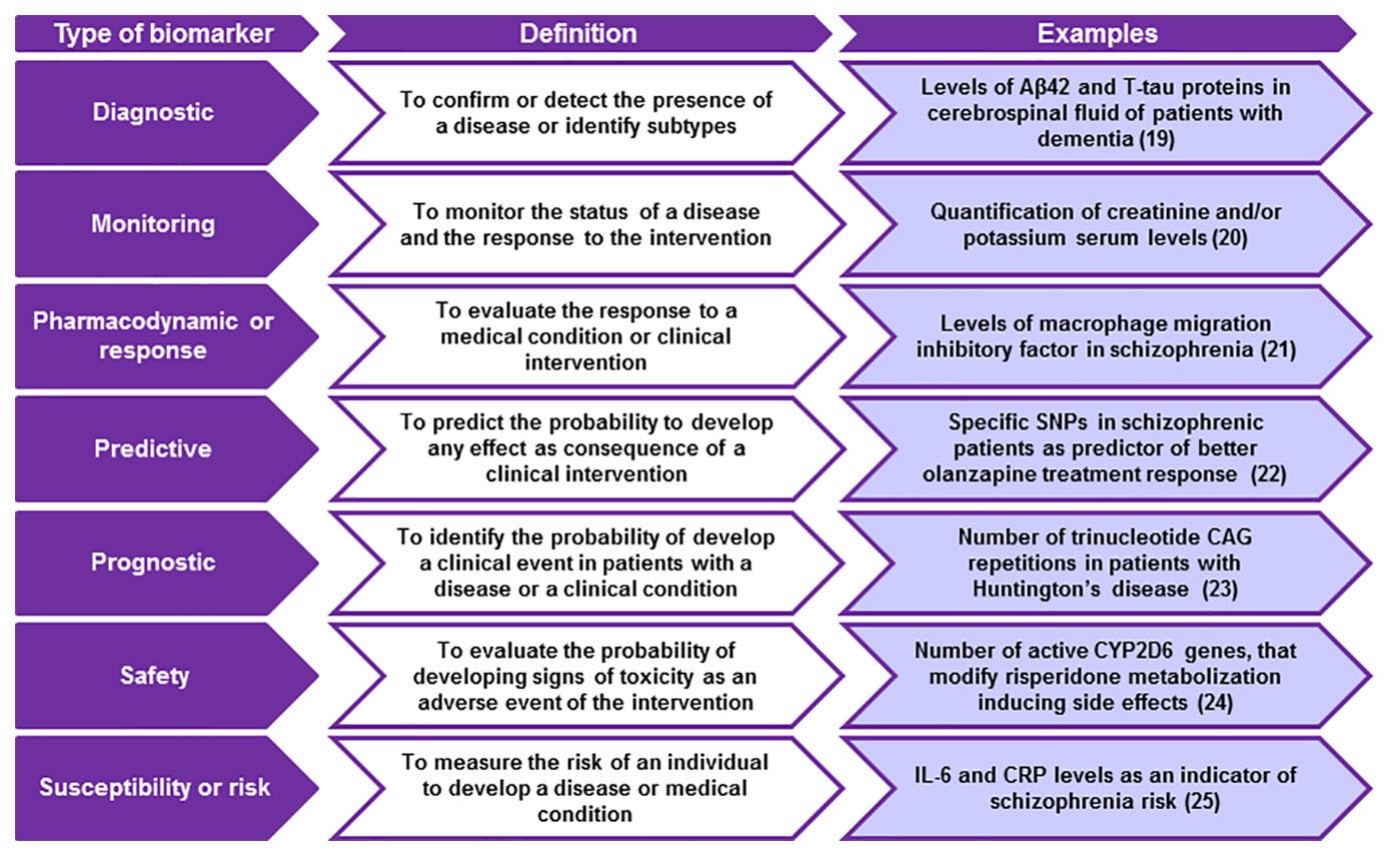
Aims and examples of the main types of biomarkers. SNPs, single nucleotide polymorphisms; CRP, c-reactive protein; IL-6, interleukin 6.

Encompasses a variety of biomarkers used to detect or confirm the presence of a disease or medical condition. This type of biomarker can be used to identify disease subtypes. The advent of the era of the precision medicine emphasizes the fact that diagnostic biomarkers are useful not only to identify patients with a disease, but also to redefine its classification. This is an important feature, because many diseases have subtypes with different prognosis or treatment responses. Thus, diagnostic biomarkers would contribute to improve personalized medicine increasing the effectiveness of the therapeutic response. These biomarkers may play also a critical role as *prognostic biomarkers* or *predictive treatment outcome biomarkers* (16, 17). An example could be the concentrations of Aβ42 and total tau (T-tau) in cerebrospinal fluid of patients with dementia as diagnostic biomarker for Alzheimer's disease (19) (**Figure 2**).

### Monitoring Biomarker

This category includes biomarkers that are analyzed at different time points to monitor the status of a disease or medical condition, and as a marker of the response to an intervention, including exposure to a medical product or an environmental agent (**Figure 2**) (16). Changes in biomarkers values are considered as indicators of the progression of the clinical condition and as measurements of the pharmacological response and other types of clinical interventions (17). An example of a monitoring biomarker is the elevation of serum creatinine and/or potassium concentrations after a pharmacological or medical intervention, parameters that are commonly used as an indicator of the probability to develop side effects (20). Monitoring biomarkers can be applied in different situations including clinical care or clinical trials, at the beginning of a treatment, for medical product development purposes, as a measure of the risk of developing a disease, or to evaluate the pharmacodynamics of a clinical intervention (**Table 1**).

**Table 1:** Potential role of monitoring biomarkers in neuropsychiatry.

Type of intervention	Utility	References
Clinical care or clinical trial	To evaluate patient's clinical situation during treatment or at the end of the intervention	(21)
Before treatment initiation	To detect signs and/or symptoms of a disease or medical condition as an indicative parameter of the prognosis To determine the need for prompt treatment	(22)
Medical product development	To provide information about the safety and effectiveness of a drug	(23)
Public health	To provide information about the risk of developing any disease or medical condition among the population	(24)
Pharmacodynamics studies	To provide evidences about therapeutic response	(25)

### Pharmacodynamic or Response Biomarker

Proposed to be a potential useful tool in clinical practice providing useful information for patient management. A pharmacodynamic biomarker is modified in response to a medical condition or clinical intervention, including drug treatments (16). Because of the serial nature of their assessment, this type of biomarker is frequently considered as a monitoring biomarker (20). The main utility of this biomarker is to guide the clinical management, providing crucial information for deciding whether or not to continue the treatment. Thus, pharmacodynamic biomarkers determine the progression of the treatment (26) (**Figure 2**).

Another area in which these biomarkers are of special interest is in early therapeutic drug development, being useful to establish the proof that a drug induces pharmacodynamic changes in humans related to its clinical benefit and to guide dose-response studies (18).

### Predictive Biomarker

A marker is considered as a predictive biomarker when its presence or modification allows predicting which patient or group of patients are more likely to experience an effect as consequence of being exposed to a medical product or environmental agent (16). This effect could be a symptomatic benefit, an increase in survival rates or an adverse event. These biomarkers are frequently used in randomized controlled clinical trials of new therapies. In this context, the biomarker is used to select patients for participation or to stratify them into intervention groups. If the biomarker predicts a favorable outcome, its presence may indicate a greater effect of the new therapy compared to the control therapy (20). Thus, the use of predictive biomarkers facilitates the selection of specific patients more likely to respond or not to therapy (**Figure 2**). An example of a predictive biomarker is the presence of 12 single nucleotide polymorphisms (SNPs) in Had Chinese schizophrenic population, that were correlated with greater olanzapine effectiveness (27).

### Prognostic Biomarker

Commonly used to identify the probability of developing a clinical event in patients diagnosed with a disease or medical condition (16). These events include death, disease progression or recurrence, or the development of a new medical condition. In clinical trials, prognostic biomarkers are used to identify patients more likely to develop a clinical event or disease progression, allowing to identify populations at higher risk. In this context, prognostic biomarkers are used as inclusion or exclusion criteria (17). An example of a prognostic biomarker is the number of trinucleotide CAG repetitions in patients with Huntington's disease. A high number of CAG nucleotides repetitions are correlated with a greater threshold of disease's severity (**Figure 2**) (28).

An additional utility of prognostic biomarkers is in treatment selection. They can provide information about treatment safety, guiding patient hospitalization or their entrance in intensive care units.

Several factors influence the clinical outcome, including the clinical condition severity, the effects induced by all treatments and the intrinsic characteristics of patients. Some of these characteristics may be used as a prognostic biomarker, allowing to identify patients more likely to experience a clinical event, disease recurrence or progression, and any effect (favorable or unfavorable) induced by a medical product or environmental agent (16, 20).

#### Safety Biomarker

Is any measure that can be assessed before and after the exposure to a medical intervention, or an environmental agent, allowing to identify the probability of developing signs of toxicity as an adverse event, to detect the presence of toxicity, and for monitoring its extension (**Figure 2**) (16).

For many therapies, monitoring hepatic, renal and cardiovascular functions are critical to detect toxicity ensuring the safety of the therapy under study. All safety biomarkers have in common its ability to detect or predict toxicity prior to theNBSP;onset of clinical signs and before irreversible damage. The toxicity can be determined by the detection or changes in the biomarker level.

Another usefulness of safety biomarkers is the identification of patients in which particular therapies should not be initiated because of significant safety risks. For example, genetic variations in CYP2D6 enzymes modify the response to certain drugs commonly used in psychiatry such as almost 50% of antipsychotics drugs. Alterations in the metabolism of drugs can modify its effectiveness, decreasing the response to the treatment or enhancing toxicity risk in patients (15). In case of the antipsychotic risperidone, there is a correlation between the number of active CYP2D6 genes and its cardiac toxicity. QTc interval is longer in subjects with one active CYP2D6 gene compared to patients with two. The study revealed that the number of CYP2D6 active genes was related with the corrected plasma concentration of risperidone (29). Safety biomarkers are used with this purpose in public health or in epidemiological interventions aimed to control or mitigate risk exposure.

### Susceptibility or Risk Biomarker

Is used as a risk measure to develop a disease or medical condition (8). An example is a genetic biomarker that can be detected many years or decades before the onset of clinical signs and/or symptoms of the disease (**Figure 2**) (10). Susceptibility/risk biomarkers are essential for the development of epidemiological studies aimed to evaluate the risk of developing a disease, contributing to establish preventive strategies in clinical practice. In this line, some studies suggested a potential correlation between interleukin-6 (IL-6) and C-reactive protein (CRP) levels and the risk of developing SCZ. Lower CRP levels together with the blockade of IL-6 signaling significantly increase SCZ risk, being proposed as a potential susceptibility/risk biomarkers for this neuropsychiatric disorder (30).

## Samples and Techniques Used for the Searching of Biomarkers

Biomarkers should be easily measurable, in easily accessible samples and using affordable techniques to ensure its inclusion in the routine clinical practice. Historically, plasma together with tissues obtained from biopsies were one of the most common samples used in the searching for biomarkers. Besides, based on the disease of interest, additional body fluids readily available in large amounts as urine, saliva, tear fluid, sweat, amniotic, cerebrospinal and pleural fluids, cervicovaginal secretion and wound efflux can be used for this purpose (31).

In the case of diseases of the central nervous system (CNS), such as psychiatric and neurological disorders, access to brain samples is of particular interest. In this respect, brain human post-mortem samples, usually provided by brain banks, play a crucial role. However, systematic biochemical investigations using these samples are scarce, limited and unrealistic mainly to the fact that the course of the disease cannot be monitored. In this respect, the progress of functional neuroimaging has allowed to study some neuronal functions including alterations of local cerebral flow, energy metabolism and neurotransmitter receptor density and occupation over the course of disease. Nevertheless, functional neuroimaging fails to provide information at cellular biochemistry level and the access to this technique is limited due to its high economic costs.

In this context, blood lymphocytes have gained special attention in the searching of peripheral biomarkers (32). Lymphocytes can be isolated easily from blood samples and studied on a daily basis allowing to monitor the course of the disease. This is possible due to the fact that receptor properties and transduction processes of lymphocytes are similar to those observed in the CNS. Several studies pointed out a close bidirectional interaction between the CNS and the immune system, in particular with lymphocytes (33). For instance, peripheral cytokines released by lymphocytes modify CNS functions including its autonomic control as well as neuroendocrine and behavioral responses. Besides, several evidences suggested that alterations in neurotransmitters and hypothalamic-pituitary-adrenal (HPA) axis in the CNS are concomitant with alterations in the function and metabolism of lymphocytes.

**Figure 3:**
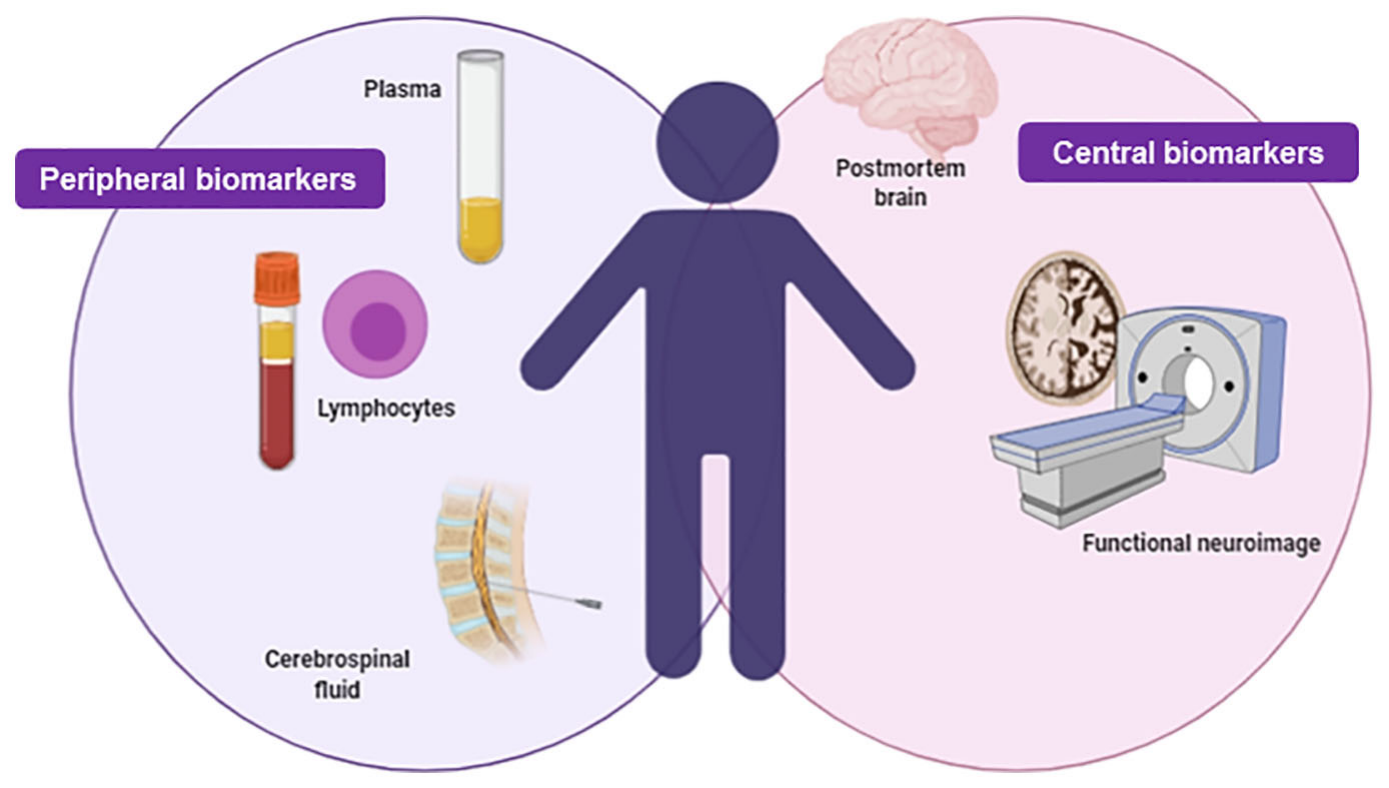
Integrative figure regarding the main samples used in the searching of biomarkers in neuropsychiatry: peripheral and central biomarkers. Created with BioRender.com.

To date, some genes such as c-fos, interleukins (IL-2, IL-4, IL-6, IL-10), nerve growth factor (NGF), brain-derived neurotrophic factor (BDNF), cannabinoid receptors, acetylcholine, GABA_A_ receptors, B_2_-adrenergic receptors, glucocorticoid receptors, mineralocorticoid receptors, D_3_ dopaminergic receptor, and serotonin receptors have been analyzed in lymphocytes from psychiatric patients, such as schizophrenic and depressive patients, with promising results as peripheral biomarkers (34–41). Thus, gene expression studies in lymphocytes of psychiatric patients at different stages of the disease, that may reflect alterations in the CNS, would allow to further characterize the mechanisms underlying the pathogenesis of the disease and may contribute to predict the pharmacological treatment response (biomarkers of treatment outcome) (**Figure 3**).

Another crucial factor for the searching of biomarkers is the techniques used. These techniques should have a high-throughput for the application of analytical data through robust dimensional data obtained in high performance tests (42). In this respect, ‘omics' technologies, including genomics, proteomics, transcriptomics, metabolomics, and epigenetics, have contributed to the rapid discovery of many potential biomarkers.

## ‘Omics’ Biomarkers and Neuropsychiatry

This section summarizes the main advantages of each ‘omics’ technology in the search of biomarkers for assessing risk, diagnosis, monitoring progression and prediction of treatment response in neuropsychiatry disorders.

### Genomic Biomarkers

**Figure 4:**
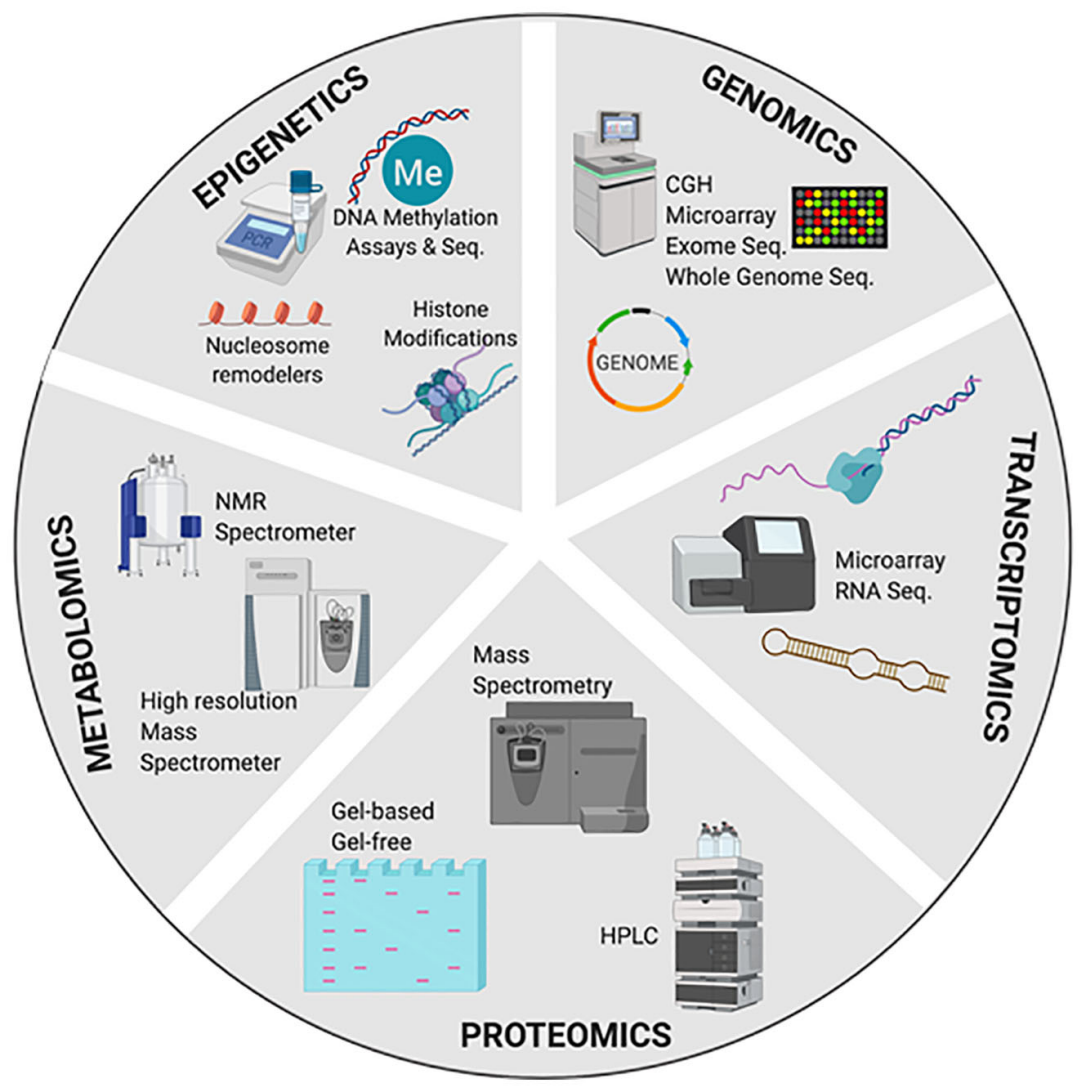
Multi-omics approach for the discovery and validation of biomarkers to probe multidimensional phases of the disease. CGH, comparative genomic hybridization; Seq, sequencing; qRT-PCR, quantitative real time PCR; qPCR, semiquantitative real time PCR; HPLC, high performance liquid chromatography; NMR, nuclear magnetic resonance spectroscopy. Created with BioRender.com.

Genomic biomarkers are expanding knowledge for the understanding of disease pathogenesis providing new targets for disease characterization, early diagnosis, and better-targeted treatment (drug discovery, drug development and adverse drug responses) to direct patients towards a more likely benefit based on their unique profile (43). According to the European Medicines Agency (EMEA), a genomic biomarker is defined as “a measurable DNA and/or RNA characteristic that is an indicator of normal biologic processes, pathogenic processes, and/or response to therapeutic or other interventions” (44). These measurable features include the expression, function and regulation of a particular gene. In the DNA, these features can be characterized by single nucleotide polymorphisms (SNPs), variability of short sequence repeats, haplotypes, deletions or insertions of (a) single nucleotide (s), copy number variations and cytogenetic rearrangements (translocations, duplications, deletions or inversions) (45). The use of genetic techniques allowed the analysis of candidate genes, genome-wide studies and polygenetic risk score analysis to understand multiple psychiatric disorders such as SCZ (46, 47). These techniques include Comparative Genomic Hybridization (CGH), microarray, exome sequencing, and whole genome sequence. Specifically, pharmacogenomics is crucial to identify genetic polymorphisms in drug-metabolizing enzymes, transporters, receptors, and other drug targets, being essential to drug discovery and drug therapy optimization [for review (48, 49)] (**Figure 4**).

Genome-wide association studies have allowed the identification of potential genomic biomarkers in different neuropsychiatric (50–52) and drug-use disorders (53). For example, the Collaborative Study of the Genetics of Alcoholism (COGA) have correlated the SNPs rs4780836 [A > C; chromosome 16:19974071 (GRCh38.p12)], rs2605140 [A > G; chromosome 17: 18253061 (GRCh38.p12)], rs11690265 [chromosome 2: C > T; chr2:27418655 (GRCh38.p12)], rs692854 [non-functional Se (FUT2) gene; alleles C > A; chromosome 19: 48706207 (GRCh38.p12)], and rs13380649 (alleles A > G; chromosome 16: 19999778 (GRCh38.p12)] with greater vulnerability and predisposition to develop alcohol use disorders (AUD) in European American and African American. Besides, the study has demonstrated that there is a correlation between these SNPs and alterations in electroencephalograms, such as lower posterior gamma, higher slow wave connectivity (delta, theta, alpha), higher frontal gamma ratio and higher beta correlation in the parietal area of patients with AUD (54).

In bipolar disorder (BD), the SNP rs17026688 in the gene encoding glutamate decarboxylase-like protein 1 (GADL1) has been associated with the response to lithium in Chinese patients (55). This SNP has been related to immune dysfunctions in BD patients, such as higher percentages of total T cells, CD4^+^T cells, activated B cells and monocytes. Besides, treatment of BD patients-derived peripheral blood mononuclear cells (PBMCs) with lithium *in vitro* increases the immune response (CD4^+^ cells). These findings suggest that the immune imbalance might not only be a biomarker for diagnosis but also a biomarker of the disease progression and therapeutic response in BD

In addition, a large study carried out through Europe, North America and Australia identified 30 genome-wide significant loci for BD in patients of European descent. These loci contain genes that encode for neurotransmitters transporters, synaptic components, and ion channels, including calcium voltage-gated channel subunit alpha1 C (CACNA1C) and other voltage-gated calcium channel genes. Among the 30 loci identified in BD patients, eight have also been described in SCZ patients (56–58); however, conditional analyses performed in this study suggested that BD and SCZ associations are independent for three of the eight shared loci, providing information that may be useful for understanding the genetics mechanisms underlying these psychiatric disorders that in some cases present symptoms in common that make its diagnosis difficult. Furthermore, the BD subtype polygenic risk score analyses performed in the study supported the nosological distinction between bipolar I (BD1) and bipolar II disorder (BD2) and the importance of psychosis beyond DSM subtypes. One limitation of the study is the genetic heterogeneity of the samples that may contribute to inconsistent replication in some of the results (59).

Besides, DNA genomic biomarkers as a useful indicator of the state of the disease (severity) also presented relevant consequences for the clinical management of neuropsychiatric diseases. In this respect, a recent study revealed a close relationship between the SNPs rs1360780 in the FK506-binding protein 5 (FKBP5) gene and rs17689918 in the corticotrophin-releasing hormone receptor 1 (CRHR1) gene and greater severity of the disease in posttraumatic stress disorder (PTSD) patients (60).

### Transcriptomic Biomarkers in Neuropsychiatry

The transcriptome is defined as the complete set of all RNA molecules in one cell or a population of cells at a specific developmental stage or physiological condition (61). Thus, transcriptome is dynamic and reflects the cellular state. Measuring the expression of an organism’s genes as a snapshot in different tissues, conditions, or time points provides information on how genes are regulated and would contribute to a better understanding of human diseases and their pharmacological treatment, allowing to identify potential therapeutic biomarkers when variations in treatment outcomes occur (62, 63). Although first studies for transcriptome began in the early 1990s, technological advances have spread throughout this time. There are two key contemporary techniques in the field: microarrays, which quantify a set of predetermined sequences, and RNA sequencing (RNA-Seq), which uses high-throughput sequencing to capture all RNA sequences (63).

For instance, transcriptomics studies identified that the efficacy of antidepressants is related to gene expression changes at transcriptome-wide scale. In a microarray study, alterations of MMO28 and KXD1 genes encoding for matrix metallopeptidase 28 and KxDL motif-containing protein 1, respectively, were associated with better response to nortriptyline in depressive patients (64). This data could contribute to improve the characterization of the molecular pathways underlying the efficacy of antidepressants.

In addition, a clinical study associated new miRNAs (miR-146a-5p, miR-146b-5p, miR-24-3p, and miR-425-3p) with the effectiveness of antidepressant drugs such as duloxetine, escitalopram, and nortriptyline, in patients with MDD. These miRNAs are ubiquitously expressed and highly correlated in blood and in brain tissue, playing an important role in the regulation of the mitogen-activated protein kinase (*MAPK*) and *Wnt* signaling pathways, closely related with stress response and MDD (65). Interestingly, an additional study revealed that MDD patients responders to antidepressant treatment present a significant reduction of miR-1202 baseline levels compared to non-responders. Moreover, miR-1202 increases as the efficacy of the antidepressant treatment is observed (66).

Besides, a total of 25 miRNAs have been modified in the amygdala of rats exposed to the learned helpless animal model of depression being the miR-128-3p the most affected. Also, a reduction of *Wnt* signaling genes has been also detected. Accordingly, an increase of miR-128-3p expression along with a significant downregulation of key target genes from *Wnt* pathway signaling (WNT5B, DVL, and LEF1) has been identified in the AMY of MDD patients (67).

Especially, RNA studies provide promising results in the searching for biomarkers of suicide. Alterations of RNA editing on the cyclic nucleotide phosphodiesterase (PDE, particularly PDE8A involved in the hydroxylation of cAMP and cGMP) were found in the dorsolateral prefrontal cortex (DLPFC) and the anterior cingulate cortex (ACC) of suicide completers. These alterations have been proposed as a biomarker of risk for attempting suicide in patients with depressive symptoms (68).

Other example of the potential role of these biomarkers in neuropsychiatry is a genome-wide expression study in patients who met the DSM-IV criteria for methamphetamine dependence. The results revealed that treatment with topiramate significantly modified the gene expression of specified genes GRINA, PRKACA, PRKCI, SNAP23 and TRAK2 involved in severe pathways underlying drug addiction and other relevant physiological functions, including neuronal function/synaptic plasticity, signal transduction, cardiovascular function, and inflammation/immune response (69).

Likewise, the microRNA-124 (miR-124) and microRNA-181 (miR-181) were pointed out as potential biomarkers for cocaine use disorder (CUD) (70). The study revealed that these two microRNAs were upregulated in the blood samples of females CUD compared with healthy female controls.

### Proteomic Biomarkers in Neuropsychiatry

Proteomics approaches using blood, plasma or serum constitutes a highly desired method for biomarker profiling of psychiatric disorders, due to the fact that these biological samples are used for routine diagnostic analyses in clinical practice, making easier to obtain samples. Besides, in neuropsychiatry, the cerebrospinal fluid (CSF) is a sample of particular interest for the identification of potential proteomic biomarkers due to its proximity to the brain. Although its collecting is very complex, due to the invasive procedure involved, it contains much less proteins than plasma. Thus, the “buffering” of protein composition is much weaker and tend to lead in a reduction of chances to identify potential proteomic biomarkers.

In proteomics, the separation of proteins using gel-based or gel free techniques, commonly followed by mass spectrometry are the mainly techniques used. The strategies for obtaining biological samples are diverse, but it is recommended to reduce the complexity of the sample, and sometimes to employ enrichment techniques improving the levels of certain subcellular fractions of interest or for specific types of proteins (glycoproteins, membrane, secreted, nuclear matrix and phosphorylated proteins) (71).

Diagnostic complications and timely treatment in neuropsychiatric disorders are frequent. Such is the case of SCZ, diagnosed by certain signs and symptoms but not by measurable and identifiable biological characteristics. In this respect, proteome studies carried out in blood plasma, serum and postmortem brain tissue from SCZ patients identified alterations in proteins that play a significant role in neuronal transmission and synaptic function, calcium homeostasis and signalling, energy metabolism, oxidative stress, cytoskeleton and in immune system and inflammation. These proteins have been proposed as biomarker candidates for prognosis, diagnosis, and medication monitoring in SCZ (72, 73). One of these proteins is zinc finger protein 729 that was found significantly down-regulated in patients with SCZ compared to healthy individuals and patients diagnosed with depression or BD (74). Another example is the study that showed reduced plasma levels of glia maturation factor beta (GMF-β), the brain-derived neurotrophic factor (BDNF), and the 115-kDa isoform of the Rab3 GTPase-activating protein catalytic subunit (RAB3GAP1) in SCZ patients. These biological markers have been proposed as potential biomarkers in this pathology (75).

Besides, the acetyl-l-carnitine (LAC) has been proposed as a proteomic biomarker in MDD. LAC plays an important role in several behavioral features. The reduction of LAC concentrations was associated with abnormal hippocampal glutamatergic function and plasticity. Such alterations suggested that the degree of LAC deficiency was directly proportional with the severity, the age of MDD onset, and the clinical history of treatment-resistant depression (TRD). These findings suggest that LAC may be useful as a diagnostic and prognosis biomarker for MDD (76).

Recently, neurofilaments light chains (NF-L) have been proposed as potential biomarkers for neuronal damage in certain psychiatric diseases. In the plasma of female patients affected by anorexia nervosa, levels of NF-L were significantly elevated, being associated with the neuronal damage observed in AN patients, that partially normalizes with weight recovery (77). An additional study pointed out the potential role of NF-L as a discriminative biomarker between primary psychiatric disorders and neurodegenerative clinical conditions with wide-ranging of behavioral, psychiatric, and cognitive symptoms (78). Interestingly, a reduction of NF-L has been identified in the hippocampus of rodents exposed to an animal model of depression (inescapable stress). In this study, treatment with valproic acid reduces depressive-like behavior and reverses NF-L reduction (79). Besides, elevated concentrations of NF-L have been observed in the CSF of BD patients. Authors demonstrated that there is a positive correlation between CSF NF-L levels and the response to antipsychotics and lithium (80). However, another prospective study failed to observe any association between the high baseline NF-L levels in CSF and clinical outcomes in BD (81). Further studies are needed to identify the role of neurofilaments as biomarkers for psychiatric disorders.

In conclusion, to consider a potential proteomic biomarker, it is necessary to evaluate its sensitivity, specificity and positive or negative predictive values upon the disease of interest (31). In this respect, modern proteomics workflows that enable high throughput studies with large cohorts of well-defined samples represent the opportunity to solve the limited reproducibility of past proteomic workflows (73).

### Metabolomics Biomarkers in Neuropsychiatry

Metabolomics biomarkers for drug development are growing. This technology focuses on the presence of small molecules metabolites in various complex matrices like CSF, blood, urine, saliva, and other human fluids. The metabolome is inherently more dynamic and time sensitive than proteome and genome, providing a direct functional measure of cellular activity and physiological status (82, 83). Changes in metabolome are the consequence of the interaction between lifestyle, environmental, genetic, developmental, and pathological factors. Consequently, metabolomics are of particular interest because, in contrast to genomics, captures the dynamic nature of the disease, and in contrast to proteomics, metabolomics measure the final products produced by complex interactions between proteins, signalling cascades and cellular environments.

Metabolomics biomarkers are not characterized by one single metabolite. Rather, they are a set of correlated metabolites defining a specific state of disease or the response to a clinical or pharmacological intervention (84). Currently, gas chromatography-mass spectrometry (GC-MS), liquid chromatography-mass spectrometry (LC-MS), and nuclear magnetic resonance (NMR) are the main types of analytical platforms used in the searching for metabolomics biomarkers.

Several studies have focused on the identification of potential metabolomics biomarkers in different psychiatric diseases [for review see (85–88)]. For example, urinary metabolomics have been proposed to be potential useful tools for the identification of pathways that may be involved in the mechanism of action of specific treatments. Such is the case of the study in which 77 urinary metabolites were identified in children with autism spectrum disorder treated with sulforaphane, a supplement that significantly improved the social responsiveness. Some of these metabolites play a role in the regulation of neurotransmitters, hormones, oxidative stress, amino acid/gut microbiome, and sphingomyelin metabolism (89).

Recently, a study conducted in patients with symptoms of depression, tried to found a predictor or a biological correlation of depression recovery after the administration of certain antidepressants including escitalopram, bupropion-escitalopram or venlafaxine-mirtazapine combinations. An increase of phosphatidylcholine C38:1 baseline plasma concentrations was associated with poorer outcome in patients. In contrast, an increased ratio in hydroxylated sphingomyelins after 8 weeks of treatment was linked to symptoms recovery (90).

However, few metabolomics biomarkers, especially in neuropsychiatry, have passed the regulatory standards for their use in clinical practice, mainly due to the lack of robust assays for the routine quantification of potential biomarkers and the heterogeneity of studies. The reduced sample size, particularly of some clinical subgroups, and the limited quantitative power of current mass spectroscopy technology, hampered the identification of robust metabolomics biomarkers, making necessary their validation trough additional assays. Besides, the complexity of samples, such as urine or blood, also contributes to that reality. In this respect, the use of chromatographic techniques for separation is needed to reduce the potential interferences associated with the complexity of human samples (91).

### Epigenetic Biomarkers

Dynamic variations in the structure of chromatin, which do not change the sequence of DNA itself but modified the expression of genes, have been paid attention due to its potential implication in the development of human diseases, including psychiatric disorders (92, 93). Accordingly, epigenetics may provide a functional interface between genotype, environmental exposure and phenotype (94).

To date, different forms of epigenetic regulation have been identified, such as direct methylation of DNA, histone modifications (as methylation and acetylation ubiquitination), exchanges of histone molecules with related isoforms, modification on chromatin by nucleosome remodelers that modify the access to DNA, and additional mechanisms like non-coding RNAs, non-genic DNAs, and differential exosome expression (95). In this way, identifying the aberrant changes in the epigenetic scenery associated to neuropsychiatry diseases and the factors that promote such alterations may allow the identification of potential new biomarkers (96).

An epigenetic biomarker is defined as “any epigenetic mark or altered epigenetic mechanism that can be measured in the body fluids or tissues defining a disease (detection); predicts the outcome of disease (prognostic), responds to therapy (predictive); monitors responses to therapy or medication (therapy monitoring) and predicts risk of future disease development (risk)” (97). So far, several techniques have been designed to analyze not only epigenetic processes at the level of specific genes but also epigenetic changes that occur in defined regions of the genome by epigenome-wide association studies. DNA methylation assays and DNA methylation sequencing are the most employed techniques, but not exclusively. Novel epigenetic techniques, such as those provided by CRISPR/Cas9 system, represent new opportunities in the searching for epigenetic biomarkers (98).

Many of the findings achieved thus far are encouraging, revealing significant associations with epigenetic modulations of genes regulating neurotransmission, neurodevelopment, and immune function in psychiatric diseases (99). One example is the hypermethylation of BDNF gene identified in brain and peripheral blood samples of MDD, SCZ and BD patients (100, 101). Another similar example is the hypermethylation of FKBP5 gene, an important modulator of stress response, detected in peripheral blood samples of PTSD patients (102). In panic disorder, hypomethylation of monoamine oxidase A (MAOA) and glutamate decarboxylases 1 (GAD1) genes have been evident in recent studies (103).

In suicide, advances in epigenetic techniques have allow to characterize epigenetic alterations in key elements of the hypothalamus-pituitary-adrenal axis (HPA-axis), neurotrophic factors, serotoninergic and GABAergic systems, that have been proposed as epigenetic biomarkers for suicide, suicide ideation and suicide attempt (104).

Interestingly, epigenetic biomarkers have been pointed out as potential biomarkers for guiding treatment. Thus, antipsychotic drugs, such as olanzapine, induced DNA methylations alterations through the brain in SCZ patients, changes related with its efficacy (105, 106). For instance, reduced response to antidepressants has been associated with the absence of methylation at a specific CpG site in exon 4 of BDNF in MDD patients (107). Consequently, BDNF exon 4 methylation, and circulating BDNF protein levels may be used together as a predictive tool to personalize treatment of MDD (108).

More interestingly, histone deacetylases (HDAC), that have been demonstrated to control epigenetic programming associated with the modulation of behaviour and cognition, appears to be crucial for reversing dysfunctional epigenetic regulation induced by early life events exposure in preclinical models (109, 110). Additional studies have supported the potential role of HDAC as promising new therapeutic targets for the treatment of MDD (111). In this context, HDAC inhibitors, alone or in combination with current antidepressant drugs, are currently being explored (112–114).

Altogether, epigenetic studies highlight the importance of epigenetic mechanisms on controlling genes or gene complexes. In neuropsychiatry, despite huge advances were achieved, there are still far for providing a clear molecular mechanism underlying these disorders and effective treatment options. The heterogeneity of the techniques and methods used, with a range in sensitivity for detecting effects (115–117); the lack of adjusting the genome-wide results to account for cell specificity (118, 119); the confounding factors such as patient's treatment, population origin and phenotypes included (105, 120); and the lack of further studies to demonstrate the concordance between brain-blood data have hampered the clinical use of epigenetic biomarkers (121).

## Summary and Conclusions

As set out in this review, there are several proteins, metabolites and genes that have been linked with certain neuropsychiatric diseases mainly due to the advance in ‘omics' technologies. However, none of them have demonstrated to be a real and useful biomarker in clinical practice.

Despite each ‘omic' presents its limitations and challenges (122–124), three essential key targets are in common to advance in the searching of biomarkers in neuropsychiatry: 1) accurate selection of the clinical population, 2) shortened sampling time and 3) standardization of procedures for sample processing. These items can be applied for any diseases, but are of special interest for psychiatric disorders. The broad spectrum of phenotypes in patients diagnosed from the same psychiatric disorder and the overlapping of some traits or clusters in different neuropsychiatric disorders, which can often make diagnosis difficult, increases the heterogeneity of the clinical population analyzed. To overcome this issue, emerged ‘omics' studies have focused on the identification of potential biomarkers for specific traits. However, the reduced number of samples analyzed per trait/phenotype has made difficult to achieve robust conclusions about the potential clinical use of the proposed biomarkers. In this respect, modern ‘omics’ workflows that enable high throughput studies with large cohorts of well-defined samples can solve this problem.

Besides, the heterogeneity of procedures for sample processing along with the differences in power and sensitivity of each ‘omics' technologies have contribute to that reality. In this respect, new ‘omics’ with better quantity power and sensitivity would contribute to find robust and realistic biomarkers.

One of the major challenges still lying ahead is the way to integrate the plethora of data obtained from each ‘omics’ to reach the holistic realization of a ‘systems biology’ understanding the biological question (125). In this context, bioinformatics tools have been designed to understand the potential of ‘omics’ technology (126).

Another concern is that current biomarker validation is a lengthy and complex process. In essence, this process includes the validation of the method, determined by the characteristics of the assay employed, and the clinical validation, to provide evidences that the biomarker is linked specifically with the disease or clinical end point under consideration. Is in this aspect in which future longitudinal integrative ‘omics’ studies can be crucial to provide a rigorously biomarkers validation ensuring its sensitivity, specificity, predictive value, and likelihood ratio, by its assessment in a large cohort (normal clinical population). It is expected that in the following years considerable breakthroughs will occur in these regards.

## Author Contributions

MG-G and JM conceived the presented idea. MG-G took the lead in writing the manuscript. FN, FS, AG, and AA-O wrote the manuscript in consultation with MG-G. All authors provided critical feedback and helped shape the research, analysis, and manuscript.

## Conflict of Interest

The authors declare that the research was conducted in the absence of any commercial or financial relationships that could be construed as a potential conflict of interest.
